# Short-term isothermic heat acclimation elicits beneficial adaptations but medium-term elicits a more complete adaptation

**DOI:** 10.1007/s00421-019-04269-5

**Published:** 2019-11-25

**Authors:** Jodie N. Moss, Freya M. Bayne, Federico Castelli, Mitchell R. Naughton, Thomas C. Reeve, Steven J. Trangmar, Richard W. A. Mackenzie, Christopher J. Tyler

**Affiliations:** 1 Department of Life Sciences, University of Roehampton, Holybourne Ave, London, SW15 4JD UK; 2 London South Bank University, Borough Road, London, SE1 0AA UK; 3 University of New England, Armidale, NSW 2351 Australia

**Keywords:** Heat strain, Acclimatisation, Endurance performance, Taper, Thermoregulation

## Abstract

**Purpose:**

To investigate the effects of 60 min daily, short-term (STHA) and medium-term (MTHA) isothermic heat acclimation (HA) on the physiological and perceptual responses to exercise heat stress.

**Methods:**

Sixteen, ultra-endurance runners (female = 3) visited the laboratory on 13 occasions. A 45 min sub-maximal (40% *W*_max_) cycling heat stress test (HST) was completed in the heat (40 °C, 50% relative humidity) on the first (HST_PRE_), seventh (HST_STHA_) and thirteenth (HST_MTHA_) visit. Participants completed 5 consecutive days of a 60 min isothermic HA protocol (target *T*_re_ 38.5 °C) between HST_PRE_ and HST_STHA_ and 5 more between HST_STHA_ and HST_MTHA_. Heart rate (HR), rectal (*T*_re_), skin (*T*_sk_) and mean body temperature (*T*_body_), perceived exertion (RPE), thermal comfort (TC) and sensation (TS) were recorded every 5 min. During HSTs, cortisol was measured pre and post and expired air was collected at 15, 30 and 45 min.

**Results:**

At rest, *T*_re_ and *T*_body_ were lower in HST_STHA_ and HST_MTHA_ compared to HST_PRE,_ but resting HR was not different between trials. Mean exercising *T*_re_, *T*_sk_, *T*_body_, and HR were lower in both HST_STHA_ and HST_MTHA_ compared to HST_PRE_. There were no differences between HST_STHA_ and HST_MTHA_. Perceptual measurements were lowered by HA and further reduced during HST_MTHA_.

**Conclusion:**

A 60 min a day isothermic STHA was successful at reducing physiological and perceptual strain experienced when exercising in the heat; however, MTHA offered a more complete adaptation.

## Introduction

Exercising for a prolonged duration in a thermally stressful environment places the body under greater physiological and perceptual strain than when exercising in temperate conditions (Galloway and Maughan 1997; Tucker et al. 2004). The greater strain often results in reduced aerobic exercise performance (Ely et al. 2008) and may even result in serious heat illness, such as endotoxemia, heat exhaustion and heat stroke (Wendt et al. 2007). Heat acclimation (HA) has been proposed as one of the most effective interventions to be incorporated into an athlete’s training programme to reduce physiological strain and improve exercise performance in hot environmental conditions (Tyler et al. 2016). Currently, the optimal HA protocol is still unknown despite a large body of research manipulating the intensity and duration of exercise, the frequency of HA exposures, and the type of HA used (Tyler et al. 2016).

Repeated exposure to thermal stress can induce beneficial adaptations that include a reduced body temperature, increases in sweating sensitivity and rate, improved cardiovascular stability, lower perceptual strain, and improved exercise economy (Tyler et al. 2016). Up to 80% of adaptations occur in the first 4–7 days of exposure (short-term heat acclimation (STHA)); however, the magnitude of adaptation appears to be greater when medium-term (MTHA; 7–14 days) and long-term (LTHA; > 15 days) HA protocols are used (Tyler et al. 2016) and not all adaptations occur over the same time course. For example, heart rate adaptations typically occur well before improvements in performance and the sudomotor responses are observed (Periard et al. 2016; Tyler et al. 2016). For heat adaptations to occur, heat stress must induce physiological strain of a magnitude above an adaptation threshold (Taylor 2014) and the magnitude of the adaptation appears dependent on the extent and frequency of the thermal strain and impulse provided. It has been proposed that the threshold for adaptation may be the attainment and maintenance of a core temperature of ~ 38.5 °C because at this temperature, sudomotor and vasomotor thermoeffector responses are challenged and heat shock proteins are expressed (Fox et al. 1964; Gibson et al. 2015a, b). It may be difficult to reach and maintain such an internal temperature using traditional constant work HA protocols, but a controlled isothermic HA protocol overcomes this issue by ensuring that the target core temperature is reached through exercise and then maintained using passive (e.g. resting) and active (e.g. exercise) heat stress.

Another potential practical benefit of isothermic HA protocols is that thermal adaptations may be achieved with shorter exercise durations and lower exercise intensities than fixed-intensity HA protocols (Gibson et al. 2015a) and, therefore, they may be appropriate during the tapering phase in training (Tyler et al. 2016). Heat adaptations are lost at a rate of approximately ~ 2.5% per day when individuals are not exposed to heat and so HA should be undertaken as close to competition as possible to minimise de-acclimation (Daanen et al. 2018); however, an overly exerting HA protocol may compromise subsequent exercise performance and health due to over-activation of the hypothalamic–pituitary–thyroid axis (Reeve et al. 2019) (resulting in increased cortisol concentrations) and/or increased permeability of the gut (leading to the translocation of endotoxins such as lipopolysaccharide (LPS) in to the blood stream) (Lim et al. 2009). Within the current literature, an isothermic HA approach has been investigated either with an absolute increase in core temperature (Magalhaes et al. 2010) or the attainment of a set thermal strain (Garrett et al. 2012; Gibson et al. 2015a). A potential limitation to using a set increase in core body temperature (e.g. + 1 °C) is that as adaptation occurs and resting core temperature lowers, individuals may not be reaching a sufficient thermal strain to elicit HA adaptations. The attainment of a set thermal strain (e.g. 38.5 °C) ensures that as adaptation occurs an adaptation stimulus continues to be provided (Taylor 2014; Tyler et al. 2016). Recent isothermic HA literature has shown that a daily 90 min isothermic HA protocol offers an adequate stimulus for heat adaptation (Garrett et al. 2012; Gibson et al. 2015a), but such a duration may be problematic to integrate in to an athlete’s preparation. Shorter (30–60 min) constant work approaches can induce beneficial heat adaptations when exercise is maintained throughout to induce the strain (Houmard et al. 1990) and so it seems prudent to suggest that maintaining the strain for a similar duration using a less-intense exercise intensity, isothermic HA regimen would also be effective and desirable to tapering athletes. It is currently unknown whether such an approach provides enough time to induce physiological and perceptual adaptations as the time spent above the thermal impulse will be considerably reduced and the total time exposed to the heat is ~ 33.3% less than in previous isothermic HA protocols (Garrett et al. 2012, 2014; Gibson et al. 2015a).

The primary aim of this study, therefore, was to investigate whether a 60 min daily isothermic (core temperature of 38.5 °C) HA regimen would reduce the physiological and perceptual strain experienced when exercising in the heat. The secondary aim was to investigate if there was a time-course effect on the physiological and perceptual adaptations and whether MTHA was more effective than STHA. We hypothesised that the isothermic HA protocol would provide a sufficient thermal impulse to induce positive changes in physiological and perceptual measurements and that these adaptations would be more complete following MTHA than STHA.

## Methods

Sixteen, non-heat acclimated, endurance runners (females = 3) participated. The mean (± SD) age, body mass, stature, body fat percentage and maximal work rate (*W*_max_) were 36.1 ± 9.1 years, 74.2 ± 9.4 kg, 176.1 ± 5.8 cm, 10.7 ± 4.9% and 302 ± 76 W, respectively. Before participation, all participants completed a health screening questionnaire and provided their fully informed, written consent to participate. The study was approved by the University of Roehampton’s ethical committee (LSC 18/228) and all procedures and protocols adhered to the guidelines of the World Medical Association (*Declaration of Helsinki*). Data were collected between March and April (Mean outside temperature ~ 6 °C) in the United Kingdom to avoid heat acclimatisation.

### Experimental design (Fig. 1)

**Fig. 1:**
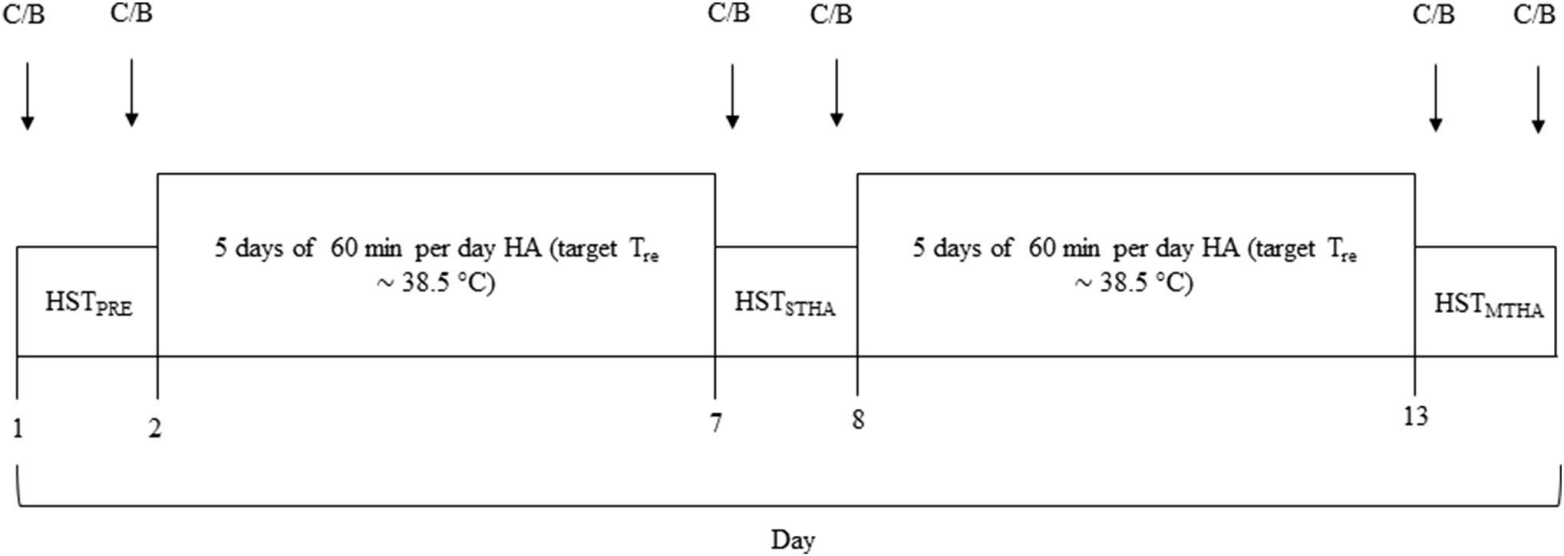
Experimental Schematic. Salivary cortisol (C) and venous blood (B) samples were collected pre- and post-HST on the first (HST_PRE_), seventh (HST_STHA_) and thirteenth (HST_MTHA_) visit. Between HST_PRE_ and HST_STHA_ and again between HST_STHA_ and HST_MTHA_, participants completed 5 consecutive days of 60 min isothermic heat acclimation during which time rectal temperature (*T*_re_) was elevated to, and maintained at ~ 38.5 °C

Participants undertook one preliminary visit (for the assessment of maximal power output) and 13 experimental visits. Participants performed a 45 min sub-maximal (40% *W*_max_) heat stress test (HST) on the first (HST_PRE_), seventh (HST_STHA_) and thirteenth (HST_MTHA_) experimental visit. Between HST_PRE_ and HST_STHA_ and again between HST_STHA_ and HST_MTHA_, participants completed 5 consecutive days of isothermic heat acclimation (HA) (60 min each visit) during which time rectal temperature (*T*_re_) was elevated to, and maintained at, ~ 38.5 °C. The environmental conditions were 40 °C and 50% relative humidity (rh) with no convective cooling for all sessions. Participants were instructed to avoid caffeine, alcohol, and strenuous exercise 24 h before all HSTs. HSTs and HA sessions were performed at the same time of day for each participant throughout the study to avoid the effects of circadian rhythm. Food intake was recorded for the 24 h prior to HST_PRE_ and participants were instructed to replicate this before HST_STHA_ and HST_MTHA_.

### Preliminary testing

Stature (Harpenden Stadiometer, Holtain Ltd, UK) and body mass (Seca, Birmingham, UK) were recorded before *W*_max_ was determined in ambient laboratory conditions (21 ± 1 °C and 55 ± 4% rh) using an incremental exercise test to volitional exhaustion (Kuipers et al. 1985), on a cycle ergometer (Monark 847E, Vansbro, Sweden). During this test, participants initially cycled at 100 watts (W) for 5 min, thereafter, work was increased by 50 W every 2.5 min until heart rate (HR) reached 160 b min^−1^, once reached, work was increased by 25 W every 2.5 min until exhaustion. The maximum work rate was calculated using the equation of Kuipers et al. (1985): *W*_max_ = *W*_com_ + ((*t*/150) × Δ*W*) [*W*_com_ is the last work rate completed; *t* is the duration (in seconds) of the final, uncompleted, stage; Δ*W* is the final load increment (typically 21 W)]. Percentage of body fat (%) was measured using whole body air displacement plethysmography method (BodPod, Cosmed, Italy).

### Heat stress tests (HSTs)

Upon arrival, a mid-flow urine sample was provided to measure urine specific gravity (USG), using a hand-held pen refractometer (Atago, pen refractometer, PEN-Urine S.G, Tokyo, Japan). All participants reported to the laboratory euhydrated (USG < 1.020). Following this, participants self-recorded nude body mass (BM) and self-inserted a rectal thermistor (REC-U-VL3-0, Grant Instruments (Cambridge) Ltd., UK) ~ 10 cm past the anal sphincter before affixing a HR monitor (Polar Electro Ltd., Kempele, Finland) to their upper torso. The rectal thermistor was connected to a portable data logger (Squirrel 2020 Series, Grant Instruments, (Cambridge) Ltd., UK). Skin temperature (*T*_sk_) was recorded continuously using wireless Thermochron iButton skin temperature data loggers (DS1922L, Thermochron iButton, USA). iButtons were attached using transparent adhesive dressing (Tegaderm, 3M Health Care, St Paul, MN) and waterproof tape (Transpore, 3M Health Care, St Paul, MN) to the sternal notch, forearm, thigh and calf muscle on the right side of the body. Mean-weighted *T*_sk_ was calculated (Ramanathan 1964) and mean body temperature (*T*_body_) was estimated (Stolwijk and Hardy 1966). Due to methodological issues, *T*_sk_, and as a result *T*_body_, data were collected from only seven participants.

Participants entered the controlled environment (40 °C, 50% rh) and rested for 2 min before baseline measurements of HR, *T*_re_, thermal sensation (TS (Young et al. 1987)), and thermal comfort (TC (Gagge et al. 1967)) were recorded and rated. Once baseline measurements were taken ,participants cycled at 40% of their *W*_max_ for 45 min, during which HR, T_re_, ratings of perceived exertion (RPE (Borg 1982)), TS and TC were measured every 5 min. One min expired air samples were collected at 14 min, 29 min and 44 min using the Douglas bag method and subsequently analysed (1400 series, Servomex, East Sussex, UK; Harvard Dry Gas Meter, Harvard Ltd., Kent, UK). To prevent further, uncontrolled per-cooling, participants drank warm (~ 37 °C) water ad libitum. The water was stored in the environmental chamber and the volume consumed was recorded. Once final measurements were recorded, participants exited the controlled environment and self-recorded a final nude BM measurement after they had towel dried. Sweat losses were determined from trial changes in BM, subtracting the weight of urine produced and adding fluid consumed (ml) during the trial.

### Heat acclimation (HA)

Participants initially repeated the same procedures as undertaken in HST_PRE_. After USG and BM were measured and a rectal thermistor was self-inserted, iButtons were placed on the same four sites and a HR strap was fitted before entering the controlled environment. Once baseline *T*_re_, HR, TS, and TC measurements were recorded after 2 min rest, participants were instructed to reach a target *T*_re_ of ~ 38.5 °C as quickly as possible and self-selected their workload and cadence accordingly. Once the target *T*_re_ had been attained, the distance cycled, and the time taken to reach the target *T*_re_ were recorded, as was the time spent at or above it. Participants then sat for the remainder of the 60 min session unless *T*_re_ fell to 38.55 °C, at which point participants resumed cycling to increase *T*_re_. During the 60 min, HR, *T*_re_, RPE, TS, and TC were recorded every 5 min and on completion of the session, nude BM was recorded post-session to estimate sweat losses. Participants drank warm water ad libitum to prevent uncontrolled per-cooling (~ 37 °C). The water was stored in the environmental chamber and the volume consumed was recorded. Due to methodological issues, *T*_sk_, and as a result *T*_body_, data were collected from only six participants. The peak intra-session strain was calculated as the peak *T*_re_ minus the starting *T*_re_ during each HA session. Peak cumulative strain was then calculated as the total strain for the 5 (STHA) and 10 day (MTHA) HA regimens.

### Salivary cortisol sample collection and analyses

Saliva samples were collected from each participant immediately upon awakening on 2 of the 5 days before HST_PRE_ (B1 and B2) to establish normal basal concentrations of cortisol and then again immediately before and after each HST. Saliva was collected by each participant chewing an absorbent swab (Salivette Cortisol, Code Blue, Sarstedt, Leicester, UK) then inserting it into a Salivette tube. All samples were centrifuged at 1000*g *for 2 min with the resulting saliva sample transferred into 2 ml Eppendorf tubes and stored in a freezer at  − 80 °C until analysis. Salivary cortisol levels were determined with a high-sensitivity (0.007 µg dL^−1^) salivary cortisol enzyme-linked immunosorbent assay (Salumetrics, State College, PA, USA) as per the manufacturer’s instructions.

### Plasma lipopolysaccharide sample collection and analyses

Venous blood was drawn from nine participants, pre- and post-HST, using a butterfly cannula that drained directly into a sterile EDTA tube before being centrifuged at 3000 rpm for 10 min at 4 °C. Plasma was extracted using pyrogen-free pipette tips into pyrogen-free microtubes (Eppendorf, Hamburg, Germany) before being frozen at  − 80 °C. Plasma concentrations of LPS were analysed using a high-sensitivity (0.04 EU ml^−1^) chromogenic limulus amoebocyte lysate end-point assay kit (Hycult Biotechnology b.v., Uden, Netherlands). Plasma samples were thawed and brought to room temperature before being diluted by 1000 times with endotoxin-free water. Fifty microliters of each sample were then transferred into the wells of pyrogen-free microplate in duplicates, followed by 50 µL of bacterial endotoxin (LAL) reagent. Optical density of the reaction was read with a microplate reader (Thermo Scientific Multiskan EX) at a wavelength of 405 nm.

### Statistical analyses

Data were analysed using SPSS (version 26, SPSS Inc.). One-way and two-way repeated measures ANOVAs were performed to determine differences between time points and trials in HST_PRE_, HST_STHA_, and HST_MTHA_, and in the first, middle, and final HA session (HA1, HA5 and HA10). Where the assumption of sphericity had been violated, the degrees of freedom were corrected using the Greenhouse–Geisser estimate. Where significant outcomes were present, post hoc tests with Bonferroni corrections were performed. The alpha level was *P* ≤ 0.05. Cohen’s *d* effect sizes were calculated for post-preload data and interpreted as follows: small effect: *d* = 0.2 to < 0.5; medium effect: 0.5 to < 0.8; large effect: *d* ≥ 0.8 (Cohen 1988). Data are presented as mean ± SD.

## Results

### Heat acclimation

There was a main effect for trial of distance (*P* = 0.025) but not duration (*P* = 0.108) cycled before the attainment of the target *T*_re_ (38.5 °C). The distance was similar between HA1 (18 ± 4 km) and HA5 (21 ± 4 km; *P* = 0.240), but greater in HA10 (23 ± 5 km) compared to HA1 (*P* = 0.018) and HA5 (*P* = 0.026). It took longer to reach the target *T*_re_ in HA10 (41.6 ± 6.4 min) than in HA1 (35.7 ± 6.1 min), but this was not statistically significant. The duration was also similar between HA1 and HA5 (36.9 ± 6.8 min) and between HA5 and HA10. Despite differences in the distance cycled, the thermal impulse to 38.5 °C was not different between HA1 (0.032 ± 0.006 °C min^−1^), HA5 (0.033 ± 0.006 °C min^−1^), and HA10 (0.036 ± 0.007 °C min^−1^) (Main effect for trial: *P* = 0.200). The peak cumulative thermal strain and impulse provided by the STHA and MTHA interventions were 9.85 ± 1.35 °C and 0.033 ± 0.0042 °C min^−1^ and 20.52 ± 2.26 °C and 0.034 ± 0.004 °C min^−1^, respectively. There were no changes in the classic physiological or perceptual markers of heat adaptation measured before or during HA1, HA5, and HA10 (Table 1).

**Table 1:** Physiological and perceptual data on the first (HA1), fifth (HA5) and tenth (HA10) heat acclimation session

	HA1	HA5	HA10
Temperature			
Resting *T*_re_ (°C)	36.92 ± 0.36	36.83 ± 0.34	36.71 ± 0.36
End *T*_re_ (°C)	38.84 ± 0.27	38.80 ± 0.25	38.85 ± 0.30
Mean *T*_sk_ (°C)	36.37 ± 0.33	36.37 ± 0.50	35.70 ± 0.58
* T*_body_ (°C)	37.58 ± 0.83	37.59 ± 0.88	37.41 ± 1.05
Total ROR (°C^.^h^−1^)	1.92 ± 0.34	1.97 ± 0.36	2.14 ± 0.44
Heart rate			
Resting HR (b^.^min^−1^)	75 ± 16	73 ± 13	72 ± 15
Mean HR (b^.^min^−1^)	135 ± 13	133 ± 13	136 ± 9
Sweat loss/fluid			
Sweat loss (L^.^h^−1^)	1.39 ± 0.41	1.75 ± 0.81	1.76 ± 0.56
Fluid consumption (L)	0.79 ± 0.20	1.01 ± 0.47	1.05 ± 0.49
Dehydration (%)	0.19 ± 0.62	0.28 ± 0.85	0.34 ± 0.86
Perceptual measurements			
Resting TC	1.1 ± 0.3	1.3 ± 0.4	1.1 ± 0.3
Mean TC	2.2 ± 0.5	2.0 ± 0.4	1.9 ± 0.4
Resting TS	4.6 ± 0.6	4.3 ± 0.9	4.3 ± 0.7
Mean TS	5.7 ± 0.6	5.6 ± 0.6	5.5 ± 0.5
Mean RPE	13 ± 2	13 ± 2	13 ± 2

Data are presented as mean ± SD

*n* = 16 for all data except for skin and mean body temperature (*n* = 6). Mean data are for the 60 min session except for RPE which is the mean of the time spent exercising only

### Heat stress test–physiological data (Table 2)

There was a main effect for trial for resting *T*_re_ (*P* < 0.001) and *T*_body_ (*P* < 0.001) with both lower in HST_STHA_ (*P* < 0.001, *d* = 1.2; *P* < 0.006, *d* = 1.6) and HST_MTHA_ (both *P* < 0.001, *d* = 1.3 and 1.6, respectively) than HST_PRE_, whereas no difference in resting *T*_sk_ was measured between HSTs (*P* = 0.243, *d* = 0.3–0.7). Resting HR was similar between HST_PRE_ and both HST_STHA_ (*P* = 0.244, *d* = 0.6) and HST_MTHA_ (*P* = 0.113, *d* = 0.7), despite a significant main effect (*P* = 0.042). There were no differences in resting *T*_re_, *T*_body_, or HR between HST_STHA_ and HST_MTHA_ (all *P* > 0.99, *d* < 0.2).

**Fig. 2:**
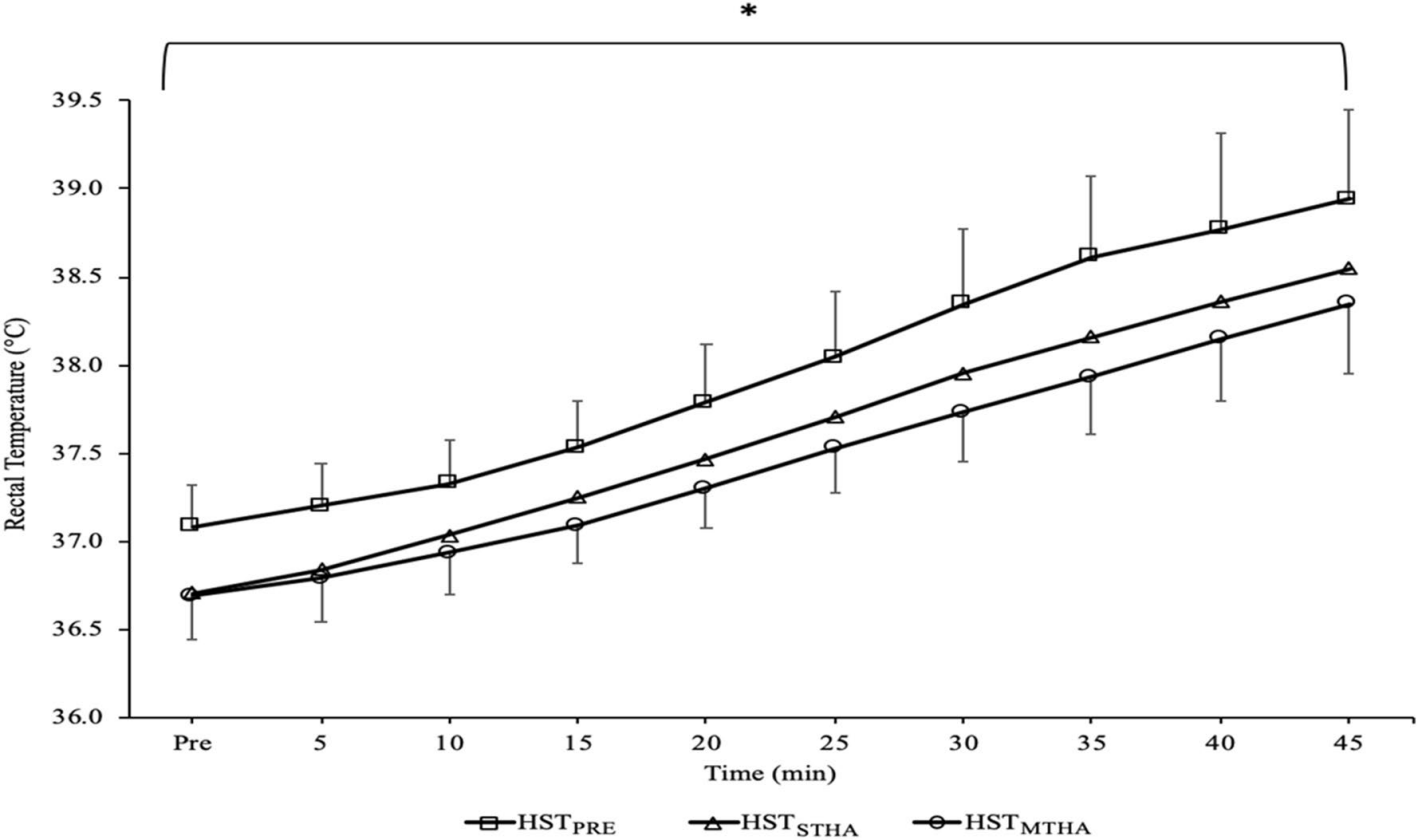
Rectal temperature (*T*_re_) on each time point during HST_PRE_, HST_STHA_, and HST_MTHA_. There were main effects of trial (*P* < 0.001) and time (*P* < 0.001) for *T*_re_, *Significant (*P* < 0.05) difference between HST_PRE_ and both HST_STHA_ and HST_MTHA._ Data mean ± SD

**Fig. 3:**
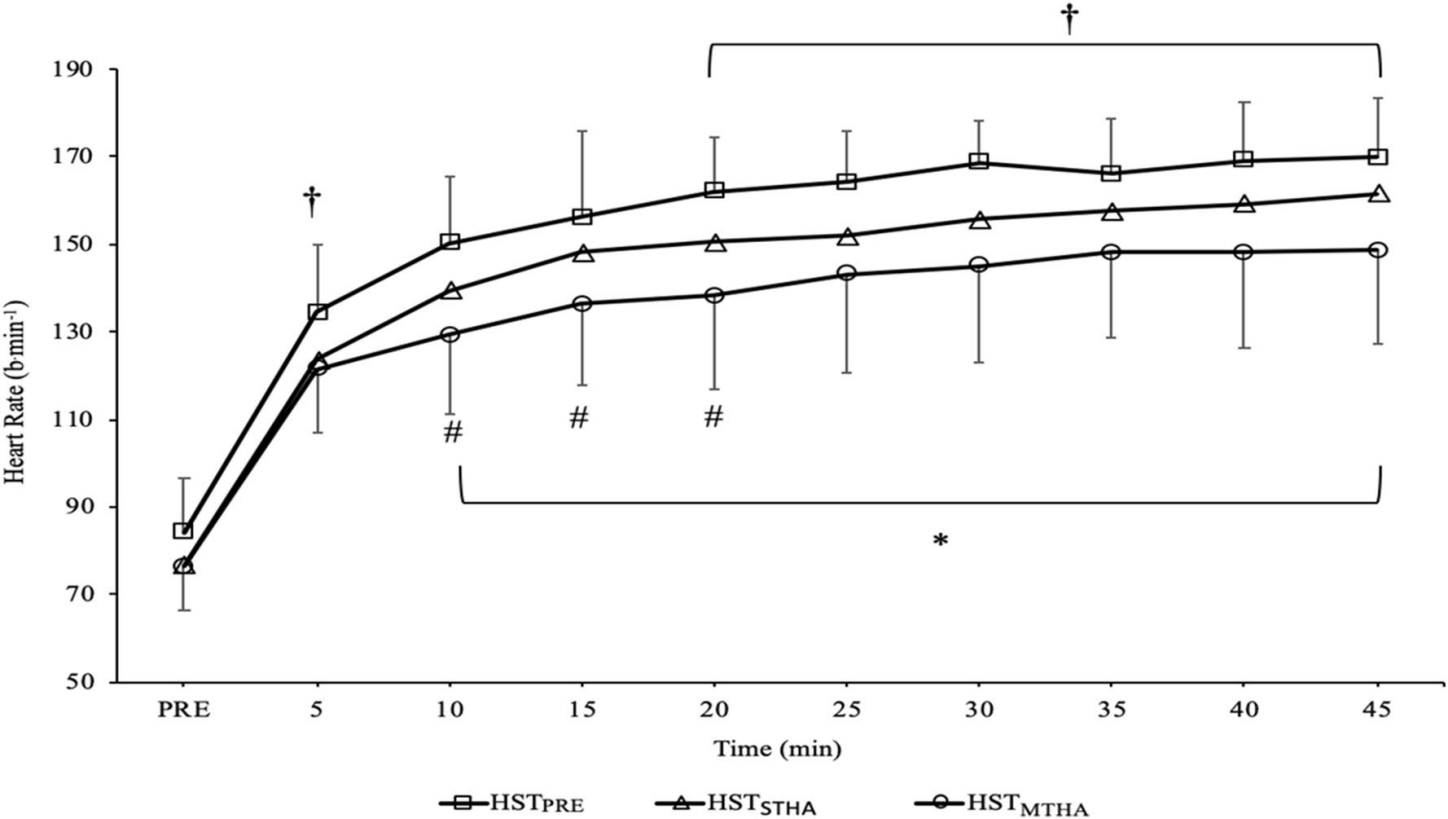
Heart rate during HST_PRE_, HST_STHA_, and HST_MTHA_. There were main effects of trial (*P* < 0.001) and time (*P* < 0.001) for HR. ^†^Significant (*P* < 0.05) difference between HST_PRE_ and HST_STHA_. *Significant (*P* < 0.05) difference between HST_PRE_ and HST_MTHA_. ^#^Significant (*P* < 0.05) difference between HST_MTHA_ and HST_STHA_. Data mean ± SD

The thermoregulatory and cardiovascular strain experienced was lower during (main effects: *P* < 0.007) and at the end (main effects: *P* < 0.005) of the HSTs performed following HA (Figs. 2 and 3). Mean *T*_re_, *T*_sk_ and *T*_body_ were higher during HST_PRE_ compared to HST_STHA_ (*P* = 0.002, *d* = 1.1; *P* = 0.026, *d* = 1.3; *P* = 0.007, *d* = 1.5, respectively) and HST_MTHA_ (*P* < 0.001, *d* = 1.4; *P* = 0.034, *d* = 1.3; *P* = 0.005, *d* = 1.5, respectively), but were similar in HST_STHA_ and HST_MTHA_ (*P* = 0.223, *d* = 0.6; *P* > 0.99, *d* = 0.1; *P* = 0.692, *d* = 0.5, respectively). The rise in *T*_re_ over the exercise bout was similar in all three HSTs (*P* = 0.292). *T*_re_ was lower at all time-points in HST_MTHA_ compared to HST_PRE_ (*P* < 0.05) and for the first 40 min in HST_STHA_ compared to HST_PRE_ (all *P* < 0.05). *T*_re_ was similar in HST_STHA_ and HST_MTHA_ at each time point (all *P* > 0.05) (Fig. 2). Mean HR was higher in HST_PRE_ than HST_STHA_ (*P* = 0.004, *d* = 0.8) and HST_MTHA_ (*P* = 0.004, *d* = 1.1) but was similar between HST_STHA_ and HST_MTHA_ (*P* = 0.074, *d* = 0.6). HR was higher in HST_STHA_ than HST_MTHA_ at 10 (*P* = 0.034), 15 (*P* = 0.018), and 20 min (*P* = 0.035) during the HST (Fig. 3).

After 45 min of exercise, *T*_re_ was not different between HST_PRE_ compared to HST_STHA_ (*P* = 0.081, *d* = 0.7) but was lower in HST_MTHA_ compared to HST_PRE_ (*P* < 0.001, *d* = 1.1), there were no differences between HST_STHA_ and HST_MTHA_ (*P* = 0.488, *d* = 0.4). *T*_sk_ and *T*_body_ was lower in both HST_STHA_ (*P* = 0.044, *d* = 1.2; *P* = 0.048, *d* = 1.3) and HST_MTHA_ (*P* = 0.018, *d* = 1.0; *P* = 0.008, *d* = 1.6) compared to HST_PRE_ but no differences were seen between HST_STHA_ and HST_MTHA_ (all *P* > 0.99, *d* = 0.41, *d* = 0.3). Final HR was higher in HST_PRE_ than HST_STHA_ (*P* = 0.012, *d* = 0.6) and HST_MTHA_ (*P* = 0.003, *d* = 1.0) but no differences were observed between HST_STHA_ and HST_MTHA_ (*P* = 0.065, *d* = 0.7). Sweat rate was similar in HST_PRE_ and HST_STHA_ (*P* > 0.99), but higher during HST_MTHA_ than HST_PRE_ (*P* < 0.001, *d* = 1.0) and HST_STHA_ (*P* < 0.001, *d* = 0.8). Fluid consumption was similar in all trials (main effect: *P* = 0.827) and so the percentage dehydration differed (main effect: *P* < 0.001), being greater in HST_MTHA_ compared to both HST_STHA_ (*P* < 0.001, *d* = 1.1) and HST_MTHA_ (*P* < 0.001, *d* = 0.8). V̇O_2_ and respiratory exchange ratio (RER) were similar between trials (main effect trial: *P* = 0.094; *P* = 0.089) and did not change over time (*P* = 0.515, *P* = 0.116).

Baseline cortisol concentrations and the concentrations prior to all HSTs were similar among the trials (*P* = 0.11). The coefficient of variation between B1 and B2 was 15 ± 6%. The within trial increase was different between trials (*P* < 0.001) being greater in HST_PRE_ (0.53 ± 0.40 µg dL^−1^; + 339 ± 284%) compared to HST_STHA_ (0.08 ± 0.29 µg dL^−1^, *P* < 0.001, *d* = 1.1; + 90 ± 183%) and HST_MTHA_ (0.04 ± 0.35 µg dL^−1^, *P* = 0.003, *d* = 1.1; + 93 ± 181%). There were no differences in the within trial change in cortisol between HST_STHA_ and HST_MTHA_ (*P* > 0.99, *d* = 0.2). There was a large degree of variation in the percentage change within trials in HST_PRE_ ( – 37 to + 730%), HST_STHA_ ( – 62 to 550%), and HST_MTHA_ ( – 63 to 580%); however, the number of participants who had an increase in cortisol concentration in their post-sample compared to their pre-sample was higher in HST_PRE_ (10/12) than HST_STHA_ (6/12) and HST_MTHA_ (6/12).

Plasma LPS levels were similar between trials before (HST_PRE_: 1.46 ± 0.57 EU ml^−1^; HST_STHA_: 1.49 ± 0.54 EU ml^−1^; HST_MTHA_: 1.52 ± 1.36 EU ml^−1^, *P* = 0.926) and after 45 min of exercise (HST_PRE_: 2.00 ± 1.65 EU ml^−1^; HST_STHA_: 1.74 ± 0.85 EU ml^−1^; HST_MTHA_: 1.72 ± 0.82 EU ml^−1^, *P* = 0.869). Within HSTs, the mean change was similar (*P* = 0.420)—0.54 ± 1.17 EU ml^−1^, 0.25 ± 1.01 EU ml^−1^ and 0.05 ± 0.38 EU ml^−1^, for HST_PRE_, HST_STHA_ and HST_MTHA_, respectively. Similar to the cortisol response, there was a large degree of variation in the percentage change within trials in HST_PRE_ (+ 50 – 218%), HST_STHA_ (+ 49 – 213%), and HST_MTHA_ (+ 29 – 402%), but unlike cortisol, LPS concentrations increased in all participants in all trials.

### Heat stress test–perceptual measurements

**Fig. 4:**
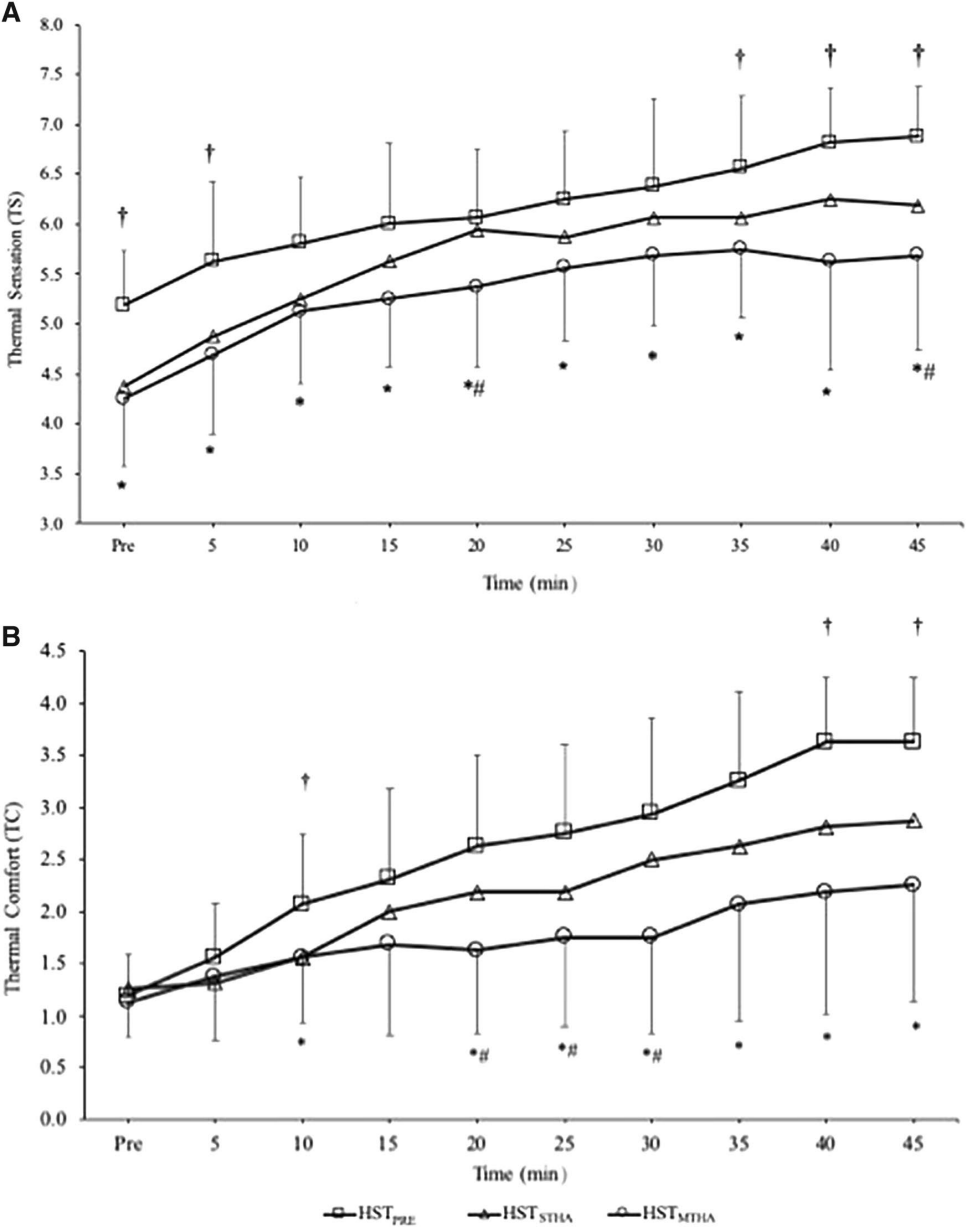
**a** Thermal sensation and **b** thermal comfort were recorded before exercise and every 5 min intervals during HST_PRE_, HST_STHA_, and HST_MTHA_. ^†^Significant (*P* < 0.05) difference between HST_PRE_ and HST_STHA_. *Significant (*P* < 0.05) difference between HST_PRE_ and HST_MTHA_. ^#^Significant (*P* < 0.05) difference between HST_MTHA_ and HST_STHA_. Data mean ± SD

There were main effects of trial and time for TS, TC, and RPE (all *P* < 0.002), with all increasing progressively throughout the HST (all *P* < 0.001), there was an interaction effect for TC and RPE (all *P* < 0.006) but not for TS (*P* = 0.248). Data are reported in Table 2. Resting TC was unaffected by HA (*P* = 0.487) but resting TS was different between trials (*P* < 0.001) being lower in HST_STHA_ (*P* = 0.014) and HST_MTHA_ (*P* = 0.002). All the reduction had occurred within 5 days of HA with no differences between HST_STHA_ and HST_MTHA_ (*P* > 0.99). During exercise, mean TS, TC, and RPE were different between trials (*P* < 0.001). TS and TC were both higher in HST_PRE_ than HST_STHA_ (*P* < 0.001, *P* = 0.037) and HST_MTHA_ (*P* < 0.002, *P* < 0.001). Both were further reduced from HST_STHA_ to HST_MTHA_ (TS: *P* = 0.031; TC: *P* = 0.030) (Fig. 4). Mean RPE was not different between HST_PRE_ and HST_STHA_ (*P* = 0.456), but was lower in HST_MTHA_ compared to HST_PRE_ (*P* = 0.006) and HST_STHA_ (*P* = 0.015). At the end of exercise, TS, TC, and RPE were all lower after HST_STHA_ (*P* < 0.001, *P* = 0.039, *P* = 0.037) and HST_MTHA_ (all *P* < 0.001) compared to HST_PRE._ Thermal sensations were rated lower at the end of HST_MTHA_ than HST_STHA_ (*P* = 0.046; *d* = 0.56) but neither TC (*P* = 0.083; *d* = 0.52) nor RPE (*P* = 0.120; *d* = 0.13) were rated differently between HST_STHA_ and HST_MTHA_.

**Table 2:** Physiological and perceptual responses to the heat stress tests (Mean ± SD)

	HST_PRE_	HST_STHA_	HST_MTHA_
Resting			
* T*_re_ (°C)	37.09 ± 0.23	36.70 ± 0.27^a^	36.69 ± 0.24^a^
* T*_sk_ (°C)	35.52 ± 0.48	35.39 ± 0.38	34.84 ± 1.24
* T*_body_ (°C)	36.76 ± 0.15	36.32 ± 0.14^a^	36.28 ± 0.18^a^
HR (b min^−1^)	84 ± 12	77 ± 12	76 ± 10
TC	1.3 ± 0.4	1.3 ± 0.4	1.1 ± 0.3
TS	5.3 ± 0.4	4.4 ± 0.8^a^	4.3 ± 0.7^a^
Mean			
* T*_re_ (°C)	37.97 ± 0.30	37.60 ± 0.28^a^	37.45 ± 0.21^a^
* T*_sk_ (°C)	37.21 ± 0.1	36.68 ± 0.1^a^	36.87 ± 0.5^a^
* T*_body_ (°C)	37.81 ± 0.74	37.25 ± 0.66^a^	37.34 ± 0.67^a^
HR (b min^−1^)	153 ± 11	142 ± 12^a^	134 ± 17^a^
V̇O_2_	2.2 ± 0.8	2.1 ± 0.7	1.7 ± 0.8
RER	0.84 ± 0.1	0.84 ± 0.1	0.74 ± 0.1
RPE	14 ± 0.6	13 ± 0.5	12 ± 0.5^ab^
TC	2.6 ± 0.7	2.1 ± 0.9^a^	1.9 ± 0.8^ab^
TS	6.2 ± 0.6	5.7 ± 0.8^a^	5.4 ± 0.7^ab^
End			
* T*_re_ (°C)	38.94 ± 0.51	38.55 ± 0.57	38.35 ± 0.39^a^
* T*_sk_ (°C)	38.11 ± 0.3	37.26 ± 0.5^a^	37.51 ± 0.5^a^
* T*_body_ (°C)	38.83 ± 0.42	38.08 ± 0.44^a^	38.19 ± 0.35^a^
HR (b min^−1^)	170 ± 13	162 ± 13^a^	149 ± 21^a^
RPE	16 ± 3	14 ± 3^a^	12 ± 3^a^
TC	3.7 ± 0.6	2.9 ± 1.3^a^	2.3 ± 1.1^a^
TS	6.8 ± 0.5	6.2 ± 0.8^a^	5.8 ± 0.9^ab^
Change *T*_re_ (°C)	1.85 ± 0.57	1.84 ± 0.65	1.66 ± 0.50
Sweat Rate (L h^−1^)	0.94 ± 0.16	1.06 ± 0.23	1.40 ± 0.30*^b^
Fluid Consumed (L h^−1^)	0.74 ± 0.15	0.70 ± 0.17	0.71 ± 0.25
Dehydration (%)	− 1.70 ± 0.30	− 1.79 ± 0.43	− 2.15 ± 0.39*^b^

*n* = 16 for all data except for skin and mean body temperature (*n* = 7)

^a^Different (*P* < 0.05) from HST_PRE_

^b^Different (*P* < 0.05) from HST_STHA_

## Discussion

The present study investigated whether a daily 60 min isothermic HA protocol provided a sufficient thermal impulse to induce the physiological and perceptual adaptations and whether there was a time-course response when comparing STHA and MTHA. The main findings of the present study are (1) an isothermic STHA protocol provides a sufficient cumulative thermal strain (9.85 ± 1.35 °C) to effectively lower physiological and perceptual strain and (2) MTHA induces further beneficial heat adaptations but only to sweat losses, final thermoregulatory strain, and perceptions of exertion, thermal strain and comfort, despite providing double the cumulative thermal strain (20.52 ± 2.26 °C).

### Physiological adaptations

The isothermic HA protocol was successful at lowering resting *T*_re_ ( − 0.38 ± 0.26 °C) and HR (8 ± 16 b^.^min^−1^) after 5 days of heat exposure. A further 5 days of heat acclimation (MTHA) did not elicit any further resting adaptations in *T*_re_ ( − 0.02 ± 0.31 °C from STHA) or HR (0 ± 9 b^.^min^−1^ from STHA). Both these responses are in accordance with previous findings that reported that these adaptations occurred at a rapid rate and were not further enhanced after longer exposure periods (Tyler et al. 2016). A lower resting *T*_re_ is an important indicator of a successful HA protocol, because it can delay the attainment of high-core body temperatures often reported to limit exercise capacity in the heat (Gonzalez-Alonso et al. 1999; Tucker et al. 2004). Our observed reductions are greater than mean changes reported in a recent meta-analysis (STHA:  − 0.17 ± 0.12 °C; MTHA:  − 0.17 ± 0.1 °C) (Tyler et al. 2016) and those reported in previous 5 day isothermic-controlled studies (Garrett et al. 2012, 2014; Neal et al. 2016). The more substantial reductions in physiological markers of heat acclimation may be due to the lower training status of our participants compared to those presented in the literature, where comparably smaller reductions in resting *T*_re_ were observed in more highly trained (mean peak power output of 375 ± 31 W) individuals (Garrett et al. 2012, 2014; Neal et al. 2016). This is likely because highly trained individuals have already developed some thermal adaptations from their long-term training history (e.g. a greater evaporative heat loss capacity and a decrease in resting core temperature) (Cheung and McLellan. 1998) which would limit the potential for a HA protocol to induce further adaptations.

The reduction in resting *T*_re_ coupled with similar (HST_PRE_: 1.85 ± 0.57 °C, HST_STHA_: 1.84 ± 0.65 °C) and lower (1.66 ± 0.50 °C) delta changes in *T*_re_ after 5 and 10 days of HA, respectively, resulted in reduced thermal strain throughout HST_STHA_ and HST_MTHA_ compared to HST_PRE_. HA has been shown to reduce the oxygen cost of exercise in the heat (Lorenzo et al. 2010); however, this is not always reported when using cycling as the mode of exercise, where the utilisation of the upper body muscles are minimal. This might explain why V̇O_2_ and RER were not altered following either STHA or MTHA in the present study. Due to the lack of change in efficiency, it seems reasonable to assume that the reduced thermal strain was due to an increase in heat loss mechanisms, as sweat rate increased after 10 days of HA, facilitating a greater heat loss through evaporative cooling. Our data support previous findings, that found sudomotor responses took longer to occur than other adaptations (Tyler et al. 2016). While local methods to access sweat rates were not used in the current study, whole body sweat rates were increased following 10 (MTHA) but not 5 (STHA) days of heat exposure. Previous results have found that HA increases sweat rate, as a result of an earlier onset of sweating at a lower core temperature and a more pronounced sudomotor thermosensitivity (Buono et al. 2018).

It is well known that cardiovascular strain can limit prolonged exercise performed under heat stress (Périard et al. 2011) and a reduced cardiovascular strain is a classic marker of an effective HA regimen (Tyler et al. 2016). In the present study, resting HR was unaffected by HA; however, the mean exercising and end of exercise HR was reduced following STHA. There was no additional benefit of a longer exposure period (MTHA) on resting and end of exercise HR; however, longer exposure time lowered HR response at certain time points during exercise. Adaptations in HR occur rapidly and are often complete within 7 days (Périard et al. 2015; Tyler et al. 2016) and data from the present study support this. With physiological measurements including stroke volume, skin blood flow and plasma volume not measured in the current study, identifying the factor that influenced this response can only be speculated. It has been previously suggested that the improved cardiovascular stability from HA is achieved through an increase in plasma volume, better maintained fluid balance, and enhanced sweating and skin blood flow responses (Périard et al. 2015, 2016; Tyler et al. 2016).

Cortisol is often used as a marker of physical and psychological strain and, as observed elsewhere (e.g. Silva et al. (2019)), cortisol concentrations increased following an initial bout of exercise in the heat (HST_PRE_). Following HA, we observed an attenuated increase—data which are in contrast to Costello et al. (2018) and Garrett et al. (2009) but in agreement with Watkins et al. (2008) who reported reductions in the session increase in cortisol after 7 days of HA. Costello et al. (2018) did not report statistical reductions in cortisol following HA but noted that there was a “trend” for the increase to be lower post-HA and so it appears that the cortisol response to exercise in the heat may be sensitive to heat adaptation. While cortisol may be a potential marker of heat adaptation, due to the variation within and between investigations, it is advisable to use the more established variables (e.g. resting core body temperature and heart rate) at present. In contrast to the cortisol response, neither STHA nor MTHA altered the LPS response to the HST. Similar observations have been reported previously by Guy et al. (2016) who also observed no effect of HA on the inflammatory response to exercise. Guy et al. (2016) postulated that their use of non-consecutive heat exposures may have provided an inadequate strain to trigger a systemic inflammatory response, but we used a more intensive HA regimen and saw comparable LPS responses. These limited data suggest that MTHA may offer some protection against endotoxemia in healthy individuals through the reduction of physiological strain and that the MTHA protocols investigated to date do not trigger an endotoxic response themselves.

### Perceptual adaptations

Participants felt more thermally comfortable and reported lower thermal sensations after 5 days of HA (STHA). An additional 5 days of HA (MTHA) had an additional beneficial effect on final thermal sensation. Both *T*_re_ and *T*_sk_ are key drivers of thermal perceptions, but in the present study, neither *T*_re_ nor *T*_sk_ continued to decline with longer exposure and so these observations do not explain why these perceptual responses continued to be improved over the HA regimen. An increase in perceived exertion (RPE) and thermal perceptions (TS, TC) have been reported to play a role in downregulating self-paced time trials (TT) when performed under heat stress to reduce the rate of heat storage well before hyperthermia is present (Tucker et al. 2004). Although not measured, it seems reasonable to suggest that a lower perceived exertion and improved perceptions of thermal strain and comfort would enable participants to select a higher exercise intensity and improve subsequent performance. Our data suggest that the 5 day isothermic STHA regimen provided a sufficient thermal stimulus to improve perceptions of strain, but MTHA (10 days) offered further benefit and so is the preferred approach.

### Limitations and practical recommendations

We cannot exclude the possibility that there was a training effect that may have occurred during HA as there was not a passive control group; however, in previous studies that included a control group, there was no reported training benefit in performance outcomes (Lorenzo et al. 2010). Additionally, the intensity and duration of exercise used in the present study was substantially lower than the participants were used to as all participants were about to take part in the Marathon des Sables, a 250 km foot-race across the Sahara desert.

Identifying an effective HA protocol that reduces the risk of overexerting an athlete so close to competition, while optimising thermal adaptations, is of current focus while athletes prepare for upcoming sporting events, including the Olympic Games in Tokyo, 2020. We did not measure whether the isothermic STHA and MTHA regimens improved subsequent exercise performance or reduced heat illness risk, but we speculate that progressive improvements would have been observed as a result of the reductions in physiological and perceptual strain as has been reported previously (Lorenzo et al. 2010). We suggest using an isothermal HA regimen during the taper phase of an athlete’s schedule and highlight that although 5 days is sufficient to induce meaningful beneficial adaptations to heat, 10 days is more effective and so should be used when possible.

## Conclusion

A 5 day 60 min isothermic HA regimen provides a sufficient thermal stimulus to elicit beneficial adaptations to reduce physiological and perceptual strain during subsequent exercise in the heat, despite providing a lower cumulative thermal strain than commonly observed in the HA literature. Most of the beneficial adaptations occurred within the STHA time-frame; however, an additional 5 days of HA (MTHA) induced further thermoregulatory, sudomotor, and perceptual adaptations and so isothermic MTHA is preferred over isothermic STHA when possible.

## Abbreviations

HA: Heat acclimation
HR: Heart rate
HST: Heat stress test
LPS: Lipopolysaccharide
MTHA: Medium-term heat acclimation
RPE: Rating of perceived exertion
STHA: Short-term heat acclimation
T_body_: Mean body temperature
TC: Thermal comfort
*T*_re_: Rectal temperature
TS: Thermal sensation
*T*_sk_: Skin temperature
*W*: Watts
